# Prolonged SARS-CoV-2 infection during autologous hematopoietic stem cell transplantation from Hodgkin's lymphoma: a case report

**DOI:** 10.11604/pamj.2023.44.133.36277

**Published:** 2023-03-16

**Authors:** Ouadii Abakarim, Fatima Ezzahra Lahlimi, Oumaima Maghnouj, Illias Tazi

**Affiliations:** 1 Department of Clinical Hematology and Bone Marrow Transplantation, University Hospital Center Mohammed VI, Faculty of Medicine and Pharmacy, Cadi Ayyad University, Marrakesh, Morocco

**Keywords:** SARS-CoV-2, autologous hematopoietic stem cell transplantation, Hodgkin’s lymphoma, chemotherapy, case report

## Abstract

Autologous hematopoietic stem cell transplantation (HSCT) for relapsed Hodgkin's lymphoma increases the risk of infection by using intensive chemotherapy. This risk is obviously ongoing given the increased virulence of severe COVID-19. We report a case of a young man with Hodgkin's lymphoma who received conditioning chemotherapy followed by autologous HSCT and tested positive for SARS-CoV-2 by polymerase chain reaction (PCR) during the early phase of aplasia with persistence of COVID-19 beyond 30 days with favorable follow-up and clinical improvement on treatment. For this type of patient with hematologic malignancy, viral infection can be fatal and strict medical precautions with isolation rules must be maintained, especially for SARS-CoV-2.

## Introduction

Immunocompromised patients with hematologic malignancies have historically been more susceptible to viral respiratory disease [1,2]. Autologous hematopoietic stem cell transplantation (HSCT) for relapsed Hodgkin's lymphoma increases the risk of infection by using intensive chemotherapy [3]. This risk is ongoing given the increased virulence of severe SARS-CoV-2 [4]. The Center for International Blood and Marrow Transplant Research (CIBMTR) has concluded that coronavirus infection in patients receiving autologous hematopoietic stem cell transplantation has a poor prognosis [5]. Here we report a case of relapsed Hodgkin's lymphoma who developed COVID-19 viral pneumonitis in bone marrow aplasia induced by conditioning followed by autologous HSCT.

## Patient and observation

**Figure 1:**
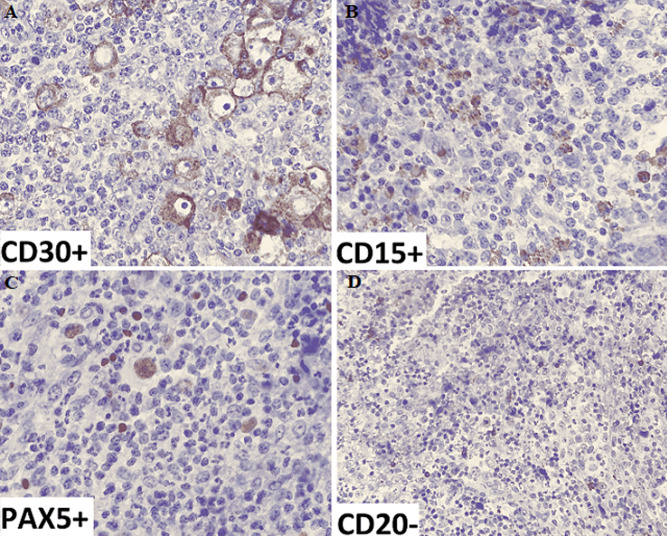
(A, B, C, D) immunohistochemistry of lymph node biopsy showing relapsed Hodgkin lymphoma in patient

**Patient information:** a 26-year-old man with relapsed Hodgkin's lymphoma (Figure 1 (A, B, C, D)), initially stage VI by pleura, who received 6 cycles of ABVD (adriamycin, bleomycin sulfate, vinblastine sulfate, and dacarbazine), 3 cycles of ICE (ifosfamide, carboplatin, etoposide), 3 cycles of DHAP (dexamethasone (steroid), high-dose cytarabine (Ara-C), cisplatin (platinum)), and 2 cycles of GEMOX (gemcitabine-oxaliplatin), in complete remission, was admitted to our bone marrow transplant unit for autologous HSCT.

**Clinical findings:** on admission, the patient was in good general condition, the clinical examination was without particularity, and the paraclinical assessment. The nasopharyngeal swab, performed by reverse transcription-polymerase chain reaction (RT-PCR), was negative for SARS-CoV-2. Myeloablative conditioning was done by the BAM protocol (Busulfan, Cytarabine, Melphalan) without incident. Hematopoietic stem cell transplantation was performed on day 0 with a total of 2.89 x 10^6^ CD34+/kg bw cells infused. On day 7, the patient presented with a fever and dry cough. Oxygen saturation was normal. A physical examination, including the chest, was normal.

**Figure 2:**
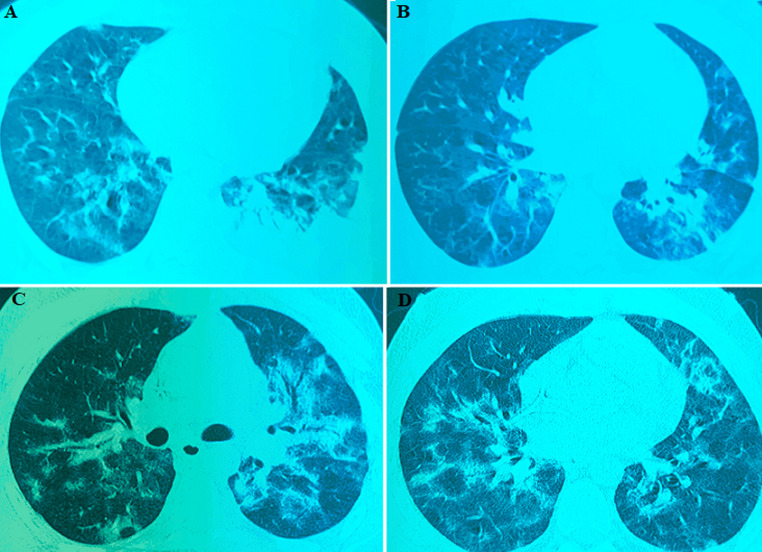
A) first chest CT scan showing minimal COVID-19 viral pneumonitis; B) second chest CT scan showing extensive COVID-19 viral pneumonitis

**Diagnostic assessment:** viral serologies were performed, and RT-PCR for SARS-CoV-2 was positive. On chest CT, the nodular ground-glass focus visible in the right dorsobasal segment was consistent with minimal COVID-19 viral pneumonitis and superimposed bacterial infection. The extent of parenchymal involvement was estimated to be between 10% and 25% (Figure 2 (A, B)). In the biological workup, C-reactive protein was elevated to 138.22 mg/L and ferritin to 4466 ng/ml. Procalcitonin levels were low at 0.52 ng/ml and D-dimer at 0.75 ug/ml.

**Therapeutic interventions:** due to the development of febrile neutropenia, the patient was initially put on: ceftriaxone 2 g/day for 3 days (day 7 to day 9) with fluconazole 400 mg on day 7, then 200mg/day for 4 days (day 8 to day 11) against oral thrush. Then, we put ceftazidime 1g x 4/day for 8 days (day 10 to day 17); ciprofloxacin 400mg x 2/day for 4 days (day 11 to day 14) in the event of persistent fever and signs of superinfection. Faced with the worsening of the infectious signs, the patient was put on amikacin 1g/day (day 12 to day 16) and voriconazole 400mg first 24h then 200mg/day for 5 days (day 12 to day 17). According to the National Guidelines on Management of Coronavirus Disease COVID-19 in Morocco, the patient was placed on azithromycin 500mg 1^st^ day and 250mg/day for 7 days (day 8 to day 14); levofloxacin 500mg x 2/day (day 14 to day 27). Corticosteroid therapy was set up with methyl-prednisolone 40mg x 2/day for 8 days (day 14 to day 21), and then 20mg x 2/day for 3 days (day 23 to day 25). The duration of the aplasia was 22 days. On day 30, the patient's condition became more complicated. He developed stage III NYHA dyspnea with dysphagia. The physical examination showed a fever of 38.5°C, polypnoea at 28 cycles/min, whitish deposits on the tongue and throat, and bilateral basithoracic crackles on auscultation. Chest CT showed a scannographic appearance consistent with COVID-19 viral pneumonitis, with extensive parenchymal involvement estimated to be between 25% and 50% (Figure 2 (C, D)). Bacterial and mycotic blood cultures were all negative. The multiplex PCR was positive for SARS-CoV-2. According to the COVID-19 Guidelines in Morocco, the patient was put on hydroxychloroquine 100mg x 2/day for 7 days, azithromycin 500mg 1^st^ day, then 250mg/day for 7 days. Heparin prophylaxis was administered during this period.

**Follow-up and outcome of interventions:** the evolution was marked by a favorable response with clinical improvement.

**Patient perspective:** during treatment, the patient was satisfied with the level of care provided to him.

**Informed consent:** the purpose of the study was explained to the patient, and informed consent was received before samples were collected. The patient was made aware that her medical records would be kept confidential.

## Discussion

The report presented here presents a patient who recovered from COVID-19 pneumonia with prolonged viral shedding during the neutropenic period of an autologous HSCT. Few studies have been published on the clinical outcomes of HSCT recipients with COVID-19 [5-7]. Patients receiving autologous HSCT have a higher risk of severe outcomes from COVID-19 in comparison with the general population. Despite this, the risk of contracting SARS-CoV-2 appears similar to the normal population [8]. Sharma *et al*. [5] studied 318 patients who underwent autologous HSCT diagnosed with COVID-19. They found that nearly half of the patients had the mild disease (49%), whereas 14% of patients had severe disease and required intensive care. At 30 days of follow-up, overall survival (OS) was 68% in allogeneic transplant recipients and 67% in autologous transplant recipients. Older age (>50 years), male gender, and patients with lymphoma had higher mortality in autologous HSCT than myeloma. There was no significant increase in mortality secondary to COVID-19 infection in patients who underwent myeloablative chemotherapy during autologous HSCT [6]. Patients who are candidates for autologous HSCT must be hospitalized for a long time. This makes them more susceptible to nosocomial infections [9]. This was the case with our patient. Thus, frequent testing of healthcare providers, including nurses and other members of the transplant team, for asymptomatic carriers of COVID-19 would be wise. The treatment of COVID-19 is controversial, and the guidelines differ. No clear recommendations exist for HSCT recipients, particularly in the early transplant setting, mainly due to the paucity of data and an unknown risk-benefit ratio [5].

Based on the recommendations of the scientific and technical committee of the national program for the prevention and control of influenza and other acute respiratory infections, the Moroccan Ministry of Health adopted a therapeutic protocol based on chloroquine and hydroxychloroquine. Approaches such as chloroquine (500 mg every 12 hours) or hydroxychloroquine sulfate (200 mg every 8 hours) for a period of 10 days, along with azithromycin 500 mg on day 1, followed by 250 mg daily from day 2 to day 7, have been suggested. In the default of clinical and/or PCR improvement, treatment will be discontinued. Lopinavir/Ritonavir at a dosage of 400mg x 2 daily for 10 days was also used as a second-line treatment. Antibiotics were not routinely used and were recommended only in case of bacterial superinfection. For patients at a high risk of thromboembolic complications, it may be important to combine low molecular weight heparin (LMWH). Patients are managed as inpatients, with strict clinical, biological, and radiological monitoring to detect any signs of deterioration at an early stage [10]. Several trials have well demonstrated the feasibility and efficacy of early management with combination therapy of hydroxychloroquine and azithromycin to prevent COVID-19-related deaths [11]. Cohorts have shown, as in the largest published outpatient series, that hydroxychloroquine-based therapy is associated with the lowest mortality rates and was not associated with severe cardiac side effects but was associated with a significant decrease in an infection fatality rate of 75% [12,13].

In contrast, those countries using hydroxychloroquine plus azithromycin or Lopinavir/Ritonavir as therapy at the onset of the epidemic have experienced a much slower dynamic in daily deaths [14,15]. In the reverse direction, numerous meta-analyses have shown the formal ineffectiveness of hydroxychloroquine alone or along with azithromycin with an increased risk of mortality [16]. The World Health Organization (WHO) does not recommend the use of hydroxychloroquine alone or along with azithromycin for COVID-19 [17]. The use of corticosteroids reduces inflammation but delays the immune reconstitution necessary for viral clearance. Corticosteroids are the most widely agreed-upon treatment in a cytokine storm [18]. A cautious compromise between immunosuppression for managed immune reconstitution and the limitation of inflammation that can damage the lungs is crucial [19]. The use of corticosteroids slightly reduces the number of deaths from all causes up to 60 days after treatment [20]. It also improves people's symptoms in patients with SARS-CoV-2 infection [18]. Our patient survived well with severe and prolonged COVID-19 under this protocol despite the presence of Hodgkin's lymphoma and post-conditioning aplasia, and controversies related to the chosen treatment.

## Conclusion

We see above the case of a patient with severe and prolonged COVID-19 after conditioning and autologous HSCT. For these types of patients with hematologic malignancies, viral infection can be severe and strict medical precautions and isolation rules should be maintained especially for SARS-CoV-2.
